# Pulsed Polarization-Based NO_x_ Sensors of YSZ Films Produced by the Aerosol Deposition Method and by Screen-Printing

**DOI:** 10.3390/s17081715

**Published:** 2017-07-26

**Authors:** Jörg Exner, Gaby Albrecht, Daniela Schönauer-Kamin, Jaroslaw Kita, Ralf Moos

**Affiliations:** Department of Functional Materials, University of Bayreuth, Universitätsstraße 30, 95440 Bayreuth, Germany; Functional.Materials@uni-bayreuth.de (J.E.); Functional.Materials@uni-bayreuth.de (G.A.); Functional.Materials@uni-bayreuth.de (D.S.-K.); Functional.Materials@uni-bayreuth.de (J.K.)

**Keywords:** Pt|YSZ system, automotive exhaust gas sensor (on-board diagnostics, OBD), aerosol deposition method (ADM), room temperature impact consolidation (RTIC), vacuum kinetic spraying

## Abstract

The pulsed polarization technique on solid electrolytes is based on alternating potential pulses interrupted by self-discharge pauses. Since even small concentrations of nitrogen oxides (NO_x_) in the ppm range significantly change the polarization and discharge behavior, pulsed polarization sensors are well suited to measure low amounts of NO_x_. In contrast to all previous investigations, planar pulsed polarization sensors were built using an electrolyte thick film and platinum interdigital electrodes on alumina substrates. Two different sensor layouts were investigated, the first with buried Pt electrodes under the electrolyte and the second one with conventional overlying Pt electrodes. Electrolyte thick films were either formed by aerosol deposition or by screen-printing, therefore exhibiting a dense or porous microstructure, respectively. For screen-printed electrolytes, the influence of the electrolyte resistance on the NO_x_ sensing ability was investigated as well. Sensors with buried electrodes showed little to no response even at higher NO_x_ concentrations, in good agreement with the intended sensor mechanism. Electrolyte films with overlying electrodes, however, allowed the quantitative detection of NO_x_. In particular, aerosol deposited electrolytes exhibited high sensitivities with a sensor output signal Δ*U* of 50 mV and 75 mV for 3 ppm of NO and NO_2_, respectively. For screen-printed electrolytes, a clear trend indicated a decrease in sensitivity with increased electrolyte resistance.

## 1. Introduction

Nitrogen oxides (NO_x_; NO and NO_2_) emissions from combustion processes may affect the environment in a serious way. Therefore, reliable monitoring devices are necessary. This creates the demand for NO_x_ gas sensors for the low ppm range that can also be operated in harsh environments like exhaust gases at high temperatures. Several types of NO_x_ sensors were already discussed in the literature, based on potentiometric, impedancemetric, or amperometric working principles (see reviews [1,2,3,4,5]), as well as new approaches like mixed potential sensors [6,7,8,9,10,11,12], or solid state gas dosimeter to measure the time dependent accumulated NO_x_ amount [13,14]. However, in the last ten years a novel technique called pulsed polarization method has been established. It was shown by a proof-of-principle-study that commercially available thimble-type lambda probes can be used to successfully detect small concentrations of NO_x_ in the ppm range [15,16,17,18]. Furthermore, the sensor mechanism during polarization and subsequent self-discharge was investigated using sensor setups consisting of 8YSZ (with 8 mol % Y_2_O_3_ stabilized ZrO_2_) substrates with platinum electrodes [19,20]. The oxygen ion conducting 8YSZ electrolyte plays a crucial role within the pulsed polarization sensing principle and provides the necessary oxygen ion transport during polarization and self-discharge. Typical NO_x_ raw emissions of diesel engines vary between 50 ppm and 2000 ppm depending on the current load. However, concentrations downstream of the catalyst are of particular interest for on-board diagnostics, with significantly reduced NO_x_ levels below 100 ppm [21].

In this study, we intended to further simplify the sensor layout, i.e., to enable a better integrability in existing (multi)-sensor setups. By using pulsed polarization measurements on 8YSZ thick films, the reported sensing mechanism is still thought to be valid. However, a significant, less complex sensor setup can be realized compared to thimble-type lambda probes. Two different layouts were investigated, both with an 8YSZ electrolyte thick film and platinum interdigital electrodes instead of 8YSZ substrates or lambda probes. The platinum electrode was either located beneath the electrolyte (buried) or above it (overlying). These 8YSZ films were applied onto alumina substrates by two different coating techniques, namely by the aerosol deposition method (which is a method to deposit dense films at room temperature directly from the powder [22]) and by screen-printing, to investigate the influence of electrolyte morphology on NO_x_ sensing properties. Aerosol deposition has already proved beneficial for a variety of gas sensors [23,24,25,26,27,28,29,30]. Furthermore, for conventional screen-printed sensors, the influence of the electrolyte resistance on the NO_x_ sensing ability is investigated by adding a passive alumina filler as well as by a variation in film thickness. This adds up to a total number of ten different sensors, that are tested for their suitability as pulsed polarization NO_x_ sensor.

## 2. Experimental

### 2.1. Sensor Preparation

**Figure 1:**
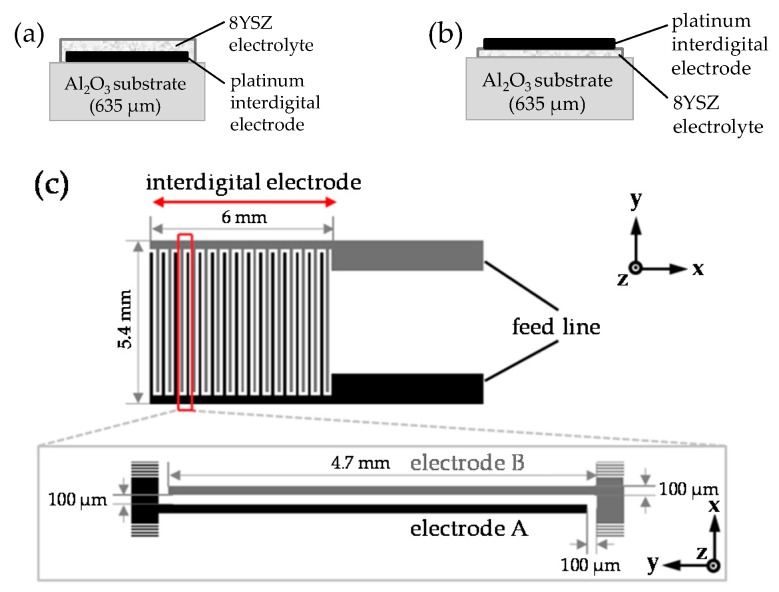
Schematic layouts for the pulsed polarization technique to detect NO_x_: cross-sectional depiction with the platinum interdigital electrode (**a**) buried between the alumina substrate and the 8YSZ electrolyte, and (**b**) overlying above the 8YSZ electrolyte. (**c**) Geometry of the platinum interdigital electrode (top view) used for both layouts.

Two different planar sensor layouts for pulsed polarization measurements were produced. In general, both layouts consist of an alumina substrate (Rubalit 708S, CeramTec, Plochingen, Germany), screen-printed and sintered platinum interdigital electrodes (Ferro 4082, Ferro Electronic Packaging Materials, Mayfield Heights, OH, USA), and an 8YSZ based solid electrolyte. For the first layout, the platinum electrode is buried between the alumina substrate and the electrolyte (Figure 1a). The second layout, however, is characterized by a similarly shaped but overlying electrodes (Figure 1b). In both cases, this interdigital electrode is made of 15 electrode fingers on each side with a width of 100 µm, a length of 4.7 mm, and a line spacing of 100 µm. Both sides of the interdigital electrode are connected by wide feed lines (Figure 1c).

Since the electrolyte is a crucial component of the sensor, the 8YSZ solid electrolyte films for each layout were applied by two different coating techniques: dense films at room temperature by aerosol deposition (AD) and porous films by screen-printing and sintering.

#### 2.1.1. Aerosol Deposited Electrolytes

**Figure 2:**
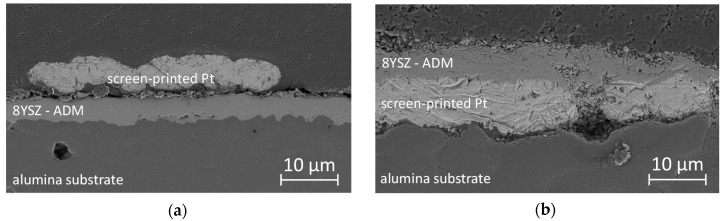
Cross-sectional SEM images of two setups. Both consist of an alumina substrate, an 8YSZ electrolyte thick film produced by aerosol deposition, and platinum interdigital-electrodes formed by screen-printing and sintering. The location of the platinum electrodes is varied: (**a**) on top of the 8YSZ film (AD 1); and (**b**) between the 8YSZ film and the alumina substrate (AD 2).

For sensors with aerosol deposited electrolytes, 8YSZ powders (Tosoh TZ-8YS, Tokyo, Japan) were ground for 4 h in a planetary ball mill using zirconia media and cyclohexane as milling liquid. Subsequently, cyclohexane was removed in a rotary evaporator and the remaining powder was dried at 200 °C. Dry powders were sieved (90 µm mesh size) to break down large, soft agglomerates, which often impede aerosol deposition. Coating was performed in a custom-made apparatus as described in [31]. For sensors with overlying platinum electrodes, the 8YSZ film was directly coated to the alumina substrate (sample is denoted as AD 1). For successful deposition, the oxygen gas flow was adjusted to 6 L/min, leading to a pressure of 200 mbar within the aerosol container and less than 0.5 mbar in the deposition chamber. Due to this pressure difference, powder particles were transported to the deposition chamber and accelerated in a slit nozzle with an orifice size of 10 mm by 0.5 mm. The substrate was kept at a standoff distance of 2 mm and moved horizontally at a speed of 1 mm/s. After cleaning with ethanol, platinum interdigital electrodes were applied onto the aerosol deposited film by screen-printing and subsequent sintering at a peak temperature of 1300 °C for 20 min. Completed sensors were also prepared for cross-sectional scanning electron microscope (SEM) imaging (Leo 1530 VP, Zeiss, Oberkochen, Germany). The obtained sensors consist of a 5 µm thick 8YSZ film with high density and strong adhesion to the alumina substrate (Figure 2a).

The screen-printed platinum has a thickness of 4 to 7 µm with several lateral holes. As a consequence, the amount of three phase boundaries between the platinum electrode, the 8YSZ electrolyte, and the surrounding gas atmosphere is strongly increased compared to dense electrodes.

For the sensor layout with a buried electrode (sample is denoted as AD 2), platinum electrodes were screen-printed directly onto the alumina substrate and sintered following the previously mentioned temperatures. Later on, 8YSZ films were formed again by aerosol deposition, now on top of the electrodes, with similar spray parameters. SEM images indicate that the 8YSZ film covers the platinum electrode completely (Figure 2b), leaving no direct access of the gas atmosphere to the electrode. The thickness of the 8YSZ film and of the platinum electrode were 6 µm and 5 to 8 µm, respectively.

#### 2.1.2. Screen-Printed Electrolytes

To produce pulsed polarization sensors with screen-printed electrolyte films, 8YSZ pastes were prepared based on Tosoh TZ-8YS powder using terpineol and ethyl cellulose as a vehicle. Furthermore, alumina got incorporated in three pastes in amounts of 5, 10, and 20 weight % to reduce the resulting electrolyte conductivity, and thus the discharge characteristics during pulsed polarization measurements were also reduced.

For sensors with overlying electrodes, screen-printing of 8YSZ or alumina-added 8YSZ pastes was conducted directly on the alumina substrates. To investigate the influence of the electrolyte thickness, sensors of pure 8YSZ electrolytes were printed twice to increase the thickness (sample SP 1). The devices were sintered by slow heating from room temperature to 400 °C with a rate of 1.6 K/min, followed by a 10 min dwell time to remove organic binders and a 3 K/min ramp up to a peak temperature of 1270 °C, which was held for 120 min. The cooling rate was 3 K/min. Platinum electrodes were screen-printed on top of the sintered 8YSZ electrolyte films and again sintered using the previously reported procedure (Section 2.1.1). For sensors with buried electrodes, 8YSZ films were screen-printed onto the alumina substrate with previous applied platinum interdigital electrodes. All prepared pulsed polarizations sensors with screen-printed, as well as aerosol deposited electrolytes are summarized in Table 1. The 8YSZ thicknesses were determined by a stylus profilometer (Mahr S2 Perthometer, Göttingen, Germany)

**Figure 3:**
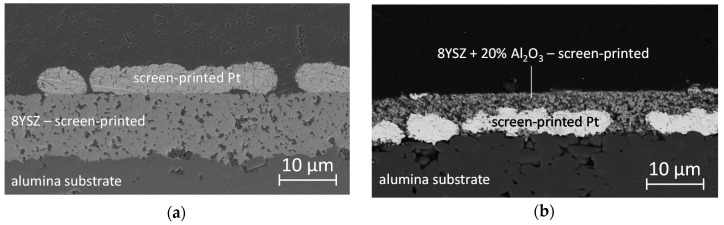
Cross-sectional SEM images of two sensor setups for the pulsed polarization technique using porous electrolytes produced by screen-printing on alumina substrates: (**a**) two times screen-printed 8YSZ films with overlying platinum electrode (SP 1); and (**b**) an electrolyte consisting of 8YSZ with incorporated Al_2_O_3_ (20 weight %) on a buried platinum electrode (SP 8).

SEM images clearly indicate the porous microstructure of the screen-printed 8YSZ films (Figure 3a) with pore sizes in the micrometer range. However, the films are still well bonded to the substrates, i.e., no cracks or delamination can be observed. Overlying platinum electrodes appear well attached to the screen-printed electrolyte, possibly because of increased film roughness due to porosity, especially compared to aerosol deposited films. The lateral electrode appearance still shows a large amount of holes. They also increase three phase boundaries. For electrolytes with added alumina, a homogeneous material mixture on the submicron scale is achieved (exemplarily shown for 20 weight % Al_2_O_3_ in Figure 3b) in electrolyte films. The pore sizes are similar to films without alumina fillers.

**Figure 4:**
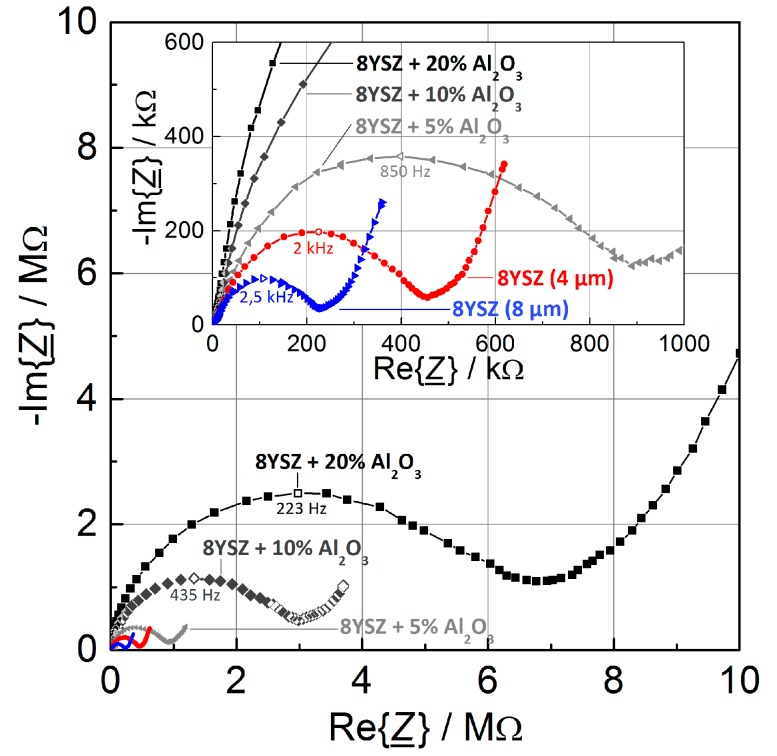
Impedance spectroscopy measurements of sensors with screen-printed electrolytes and overlying platinum electrodes. The composition of the electrolyte was varied from pure 8YSZ to additions of up to 20 weight % Al_2_O_3_ (SP 1 to SP 5). Measurements were performed in base gas (10% O_2_ and 2% H_2_O with N_2_ as a balance) at 420 °C. The hollow indicator marks the top of each semicircle and the corresponding frequency.

Electrochemical impedance spectroscopy data were recorded (Novocontrol Alpha-A Analyzer, Montabaur, Germany) in the frequency range of 10 MHz to 0.1 Hz at an amplitude *V*_rms_ of 25 mV for all sensors with overlying electrodes to quantify the change in the electrolyte film resistances. Measurement conditions were adjusted to fit later sensor operation conditions, namely a temperature of 420 °C and an atmosphere of 10% O_2_ and 2% H_2_O with nitrogen as a balance. Figure 4 shows the complex impedance in a Nyquist representation. All spectra show a semicircle at medium to high frequencies, representing the bulk conductivity, and a Warburg impedance in the low frequency range for electrode contribution.

For singly printed 8YSZ films with 4 µm thickness, the sensors show a resistance of 450 kΩ at an operating temperature of 420 °C. By doubling the thickness to 8–9 µm through an additional screen-printing step, the resistance is lowered to 240 kΩ, as intended. In contrast, additions of 5, 10, and 20 weight % alumina led to significantly higher resistances of 1 MΩ, 3.2 MΩ, and 7 MΩ, respectively.

### 2.2. Measurements

**Figure 5:**
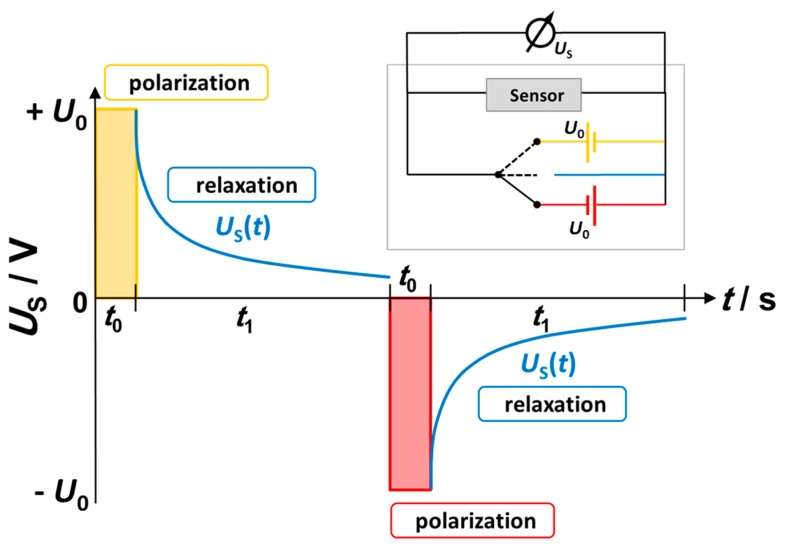
Scheme of one pulsed polarization measurement cycle with polarization and subsequent relaxation. During relaxation, the open circuit voltage is measured.

The pulsed polarization method of 8YSZ films is a dynamic measuring technique to quantify low NO_x_ concentrations in the ppm and sub-ppm range. A schematic measurement cycle is displayed in Figure 5. Each cycle consists of two polarization steps (duration *t*_0_) with alternating voltage signs, interrupted by self-discharge periods (*t*_1_).

At first, the sensor gets polarized with a potential *U*_0_ of 1.8 V for a small period *t*_0_ of 1 s. Afterwards, both electrodes were disconnected from the power source and the time-dependent self-discharge *U*_s_ (open circuit voltage) is recorded for a period *t*_1_ of 3 s. This is followed by an additional polarization with an alternated sign (−*U*_0_) for *t*_0_ and the subsequent self-discharge. The polarization voltage *U*_0_ was chosen below 2 V to avoid local decomposition of YSZ due to the removal of oxygen ions as a consequence of oxygen pumping [32,33]. Since both parts of the platinum interdigital electrode are similarly sized and are exposed to the same atmosphere, the self-discharge behavior after positive and negative pulses is symmetrical, which is in contrast to earlier studies, where a non-symmetrical behavior was observed [15] with thimble-type devices with one electrode being exposed to air. Previous investigations showed that the voltage course during relaxation is highly dependent on the presence of NO_x_ [19]. Higher concentrations result in an accelerated self-discharge, making it a well suited sensor signal. Though the exact sensor mechanism has not been fully understood yet, formation and decomposition of the platinum-oxides at the electrode surface seem to play an important role.

All measurements were conducted in a quartz tube furnace at a temperature of 420 °C. Gas atmospheres were adjusted using mass flow controllers (MFC) for N_2_, O_2_, NO, NO_2_, and water-saturated N_2_ at a total gas flow of 200 mL/min. The base gas consisted of 10% O_2_ and 2% H_2_O with N_2_ serving as a balance. For NO and NO_2_, concentrations of 24 ppm, 12 ppm, 6 ppm, and 3 ppm, in descending order, were added to the gas atmosphere for 7 min, interrupted by 7 min of base gas between each step. Also, NO:NO_2_ mixtures (volume ratio 1:1) with 48 ppm, 24 ppm, 12 ppm, and 6 ppm were tested.

## 3. Results and Discussion

### 3.1. Sensors with an Aerosol Deposited 8YSZ Electrolyte Film

**Figure 6:**
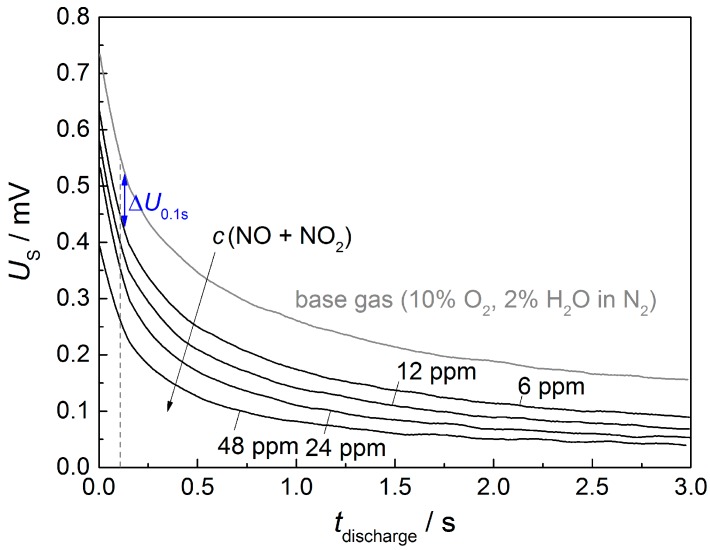
Example for a time-dependent sensor potential *U*_S_ during self-discharge in base gas and under NO_x_ concentrations between 6 and 48 ppm after alternating pulses. The sensor consists of an 8YSZ electrolyte produced by aerosol deposition and an overlying platinum electrode produced by screen-printing and sintering.

Self-discharge curves of the aerosol deposited 8YSZ film with overlying Pt electrodes in base gas and 6 ppm to 48 ppm NO_x_ are shown in Figure 6. In base gas, *U*_s_ declines from 0.75 V directly after polarization to 0.2 V at the end of the self-discharge phase after 3 s. In the presence of NO_x_, the course of the discharge curves change significantly, as expected from [17,19] with an accelerated potential decrease, especially during the first second. For example, under 48 ppm NO_x_, self-discharge seems to start at 0.4 V and declines to 0.05 V after 3 s. Therefore, it can be stated that discharge curves for this sensor follow the general sensor behavior of pulsed polarization sensors, with a similar course compared to previously tested planar sensors (based on 8YSZ substrates and two-dimensional platinum electrodes [19]).

Since the first second of self-discharge is highly sensitive to NO_x_, the sensor output signal is determined as the potential difference between base gas and currently measured atmosphere after 0.1 s of discharge:

Δ*U*_0.1s_ = *U*_s_ (base gas, *t*_discharge_ = 0.1 s) − *U*_s_ (test gas, *t*_discharge_ = 0.1 s) (1)

While sensors with overlying electrodes are showing very promising results, those with buried electrodes unsurprisingly act differently. Here, discharge curves are nearly independent of the surrounding gas atmospheres. This is a consequence of the tight sealing of platinum interdigital electrodes by the dense aerosol deposited 8YSZ films. Due to a lack of three-phase-boundaries Pt-YSZ-NO_x_, the pulsed polarization mechanism cannot come into effect, and only discharge due to ionic conductivity of 8YSZ is observed. For that reason, in the case of aerosol deposited electrolyte films, only overlying electrodes are investigated in detail.

**Figure 7:**
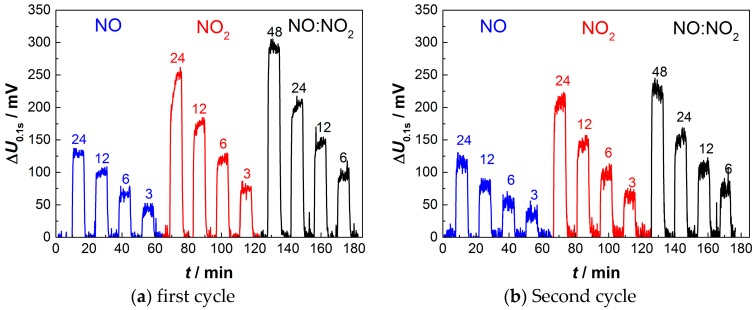
Sensor output voltage Δ*U*_0.1s_ at NO and NO_2_ concentrations between 24 ppm and 3 ppm, and NO:NO_2_ (1:1) mixtures between 48 ppm and 6 ppm, respectively, after alternating pulses during the (**a**) first and (**b**) consecutive second cycle. The sensor consists of an 8YSZ electrolyte produced by aerosol deposition and an overlying platinum electrode produced by screen-printing and sintering.

Figure 7 displays the sensor output voltage Δ*U*_0.1s_ for the programed NO, NO_2_, and mixed NO_x_ gas atmospheres with intervening base gas steps. For the first, as well as for the subsequent second measuring cycle, the sensor output signal Δ*U*_0.1s_ forms distinct plateaus when NO_x_ is present and returns to 0 mV at base gas. In general, higher concentrations of NO, NO_2_, or mixed NO_x_ (1:1) also lead to increased values of Δ*U*_0.1s_. The response towards NO_2_ is by about a factor of 1.5 to 1.75 higher compared to NO. A possible explanation could be that twice the number of electrons are necessary to reduce NO_2_ than NO. A higher consumption of charge carriers therefore could lead to the observed accelerated discharge. For mixed NO_x_ (1:1 volume mixture of NO and NO_2_), the sensor output voltage is nearly at half height between pure NO and NO_2_. In all measurements, the noise averages around 10 mV and is considerably smaller than the measured Δ*U*_0.1s_, even at small NO_x_ concentrations.

**Figure 8:**
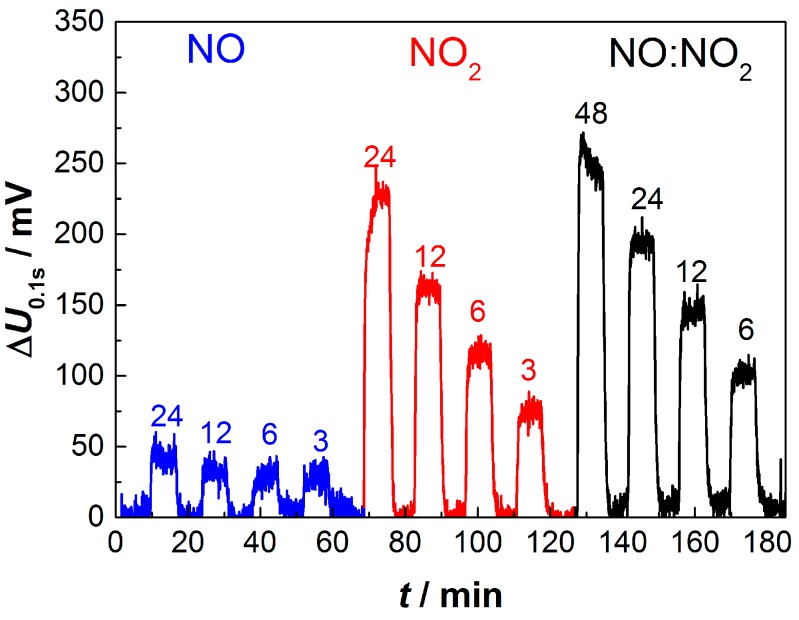
Sensor output voltage Δ*U*_0.1s_ at NO and NO_2_ concentrations between 24 ppm and 3 ppm, and NO:NO_2_ (1:1) mixtures between 48 ppm and 6 ppm, respectively, after single sign pulses. The sensor consists of an 8YSZ electrolyte produced by aerosol deposition and an overlying platinum electrode produced by screen-printing and sintering.

A small decay in the sensitivity of about 10% to 15% occurs between the first and second cycle; however, the ability to quantitatively detect NO_x_ remains. In an attempt to further increase the sensitivity towards either NO or NO_2_, the sensor is operated unidirectionally (no change in sign of polarization) instead of with alternating polarization. While the response towards NO_2_ is nearly identical compared to the alternated pulsed procedure, the influence of NO diminishes to under 50 mV even at 24 ppm (Figure 8). There is no clear reason for that. However, without alternating pulses, reduction of PtO_x_ at the first electrode and oxidation of Pt to PtO_x_ at the opposite electrode cannot occur for unidirectional pulses.

**Figure 9:**
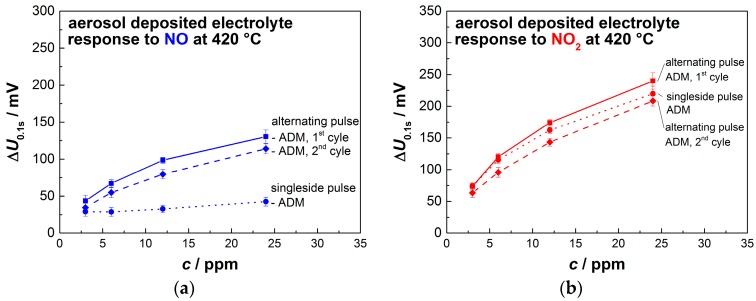
Sensor characteristics for sensors based on 8YSZ electrolytes built by aerosol deposition with overlying platinum interdigital electrodes towards small concentration of (**a**) NO and (**b**) NO_2_.

The described sensor characteristics towards NO and NO_2_ for the first and the second cycle with alternating pulses, as well as for unidirectional pulses, are summarized in Figure 9. The sensor output voltage Δ*U*_0.1s_ shows a nearly semi-logarithmic dependency on the NO_2_ concentration, independent of the applied pulsed operation mode. Response Δ*U*_0.1s_ to 3 ppm NO_2_ is always between 60 mV and 75 mV, and to 24 ppm between 210 mV and 235 mV, indicating a high and stable sensor signal. When only NO is present, Δ*U* shows a semi-logarithmic dependency only for the alternating pulsed mode, with an already described decrease in sensitivity, between the first and second measuring cycle. On the contrary, unidirectional pulses show little to no slope towards the concentration of NO. Although this behavior has not been fully understood yet, it could enable a differentiation between NO and NO_2_. A mixed operation mode could be possible—one that alternates between unidirectional pulses and alternating pulses at predefined time intervals.

### 3.2. Sensors with Screen-Printed and Sintered 8YSZ Electrolyte Films

**Figure 10:**
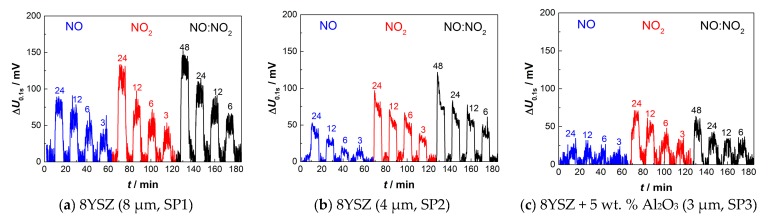
Sensor output voltage Δ*U*_0.1s_ of three different sensors at NO and NO_2_ concentrations between 24 ppm and 3 ppm, and NO:NO_2_ (1:1) mixtures between 48 ppm and 6 ppm, after alternating pulses. All sensors consist of a screen-printed 8YSZ solid electrolyte and an overlying platinum electrode also produced by screen-printing and sintering: (**a**) doubly screen-printed 8YSZ with 8–9 µm thickness; (**b**) single screen-printed 8YSZ with 4 µm thickness; and (**c**) single screen-printed mixture of 8YSZ and 5 weight % Al_2_O_3_ with 3 µm thickness

Sensor responses for devices based on screen-printed electrolytes with overlying platinum electrodes are shown in Figure 10. The doubly printed 8YSZ electrolyte with 8–9 µm thickness again provides a sensor output Δ*U*_0.1s_ that shows plateaus for all measured NO, NO_2_, and NO_x_ concentrations. The largest values of 75 mV and 115 mV occur at 24 ppm of NO and NO_2_, respectively. However, increased noise of around 25 mV and a decreased sensitivity compared to the aerosol deposited films clearly worsens the signal-to-noise ratio (SNR), yet 3 ppm of NO or NO_2_ can certainly be detected. Like those for aerosol deposited electrolytes, responses to NO are lower compared to NO_2_, but only by a factor of 1.2 to 1.5.

When using the thinner electrolyte (4 µm thickness, Figure 10b) two observations can be drawn. First, the total sensor output is smaller, especially at larger concentrations of 12 ppm and 24 ppm. Secondly, when switching from base gas to NO or NO_2_, the sensor signal Δ*U*_0.1s_ tends to overshoot, followed by a subsequent slow decline. It seems that no final value is achieved within seven minutes in NO_x_-loaded atmosphere before returning to base gas.

This indicates that a thicker electrolyte with lower resistance is preferable in order to achieve a large and stable sensor output. For electrolytes with incorporated alumina, a further decrease in sensitivity occurs (Figure 10c, with 5% Al_2_O_3_). Here, especially for NO, a determination of the concentration is not possible anymore due to the heavy noise and very small sensor response. This trend continues even more for higher alumina amounts of 10% and 20%. Here, a differentiation between base gas and even high NO_x_ concentrations is not possible anymore. The results indicate that increased electrolyte resistances deteriorate the pulsed polarization sensor sensitivity, at least at the operation temperature of 420 °C. 

The remaining sensors with buried platinum interdigital electrodes (SP6–SP8) also do not show any response to NO_x_ atmospheres, independent of their electrolyte composition. Electrolytes films produced by screen-printing show a high, probably open porosity. By that, gas atmosphere could, in theory, also get in contact with buried platinum electrodes. However, diffusion paths seem to be too long or too narrow to transport a sufficient amount of NO_x_ from the gas atmosphere to the electrodes.

**Figure 11:**
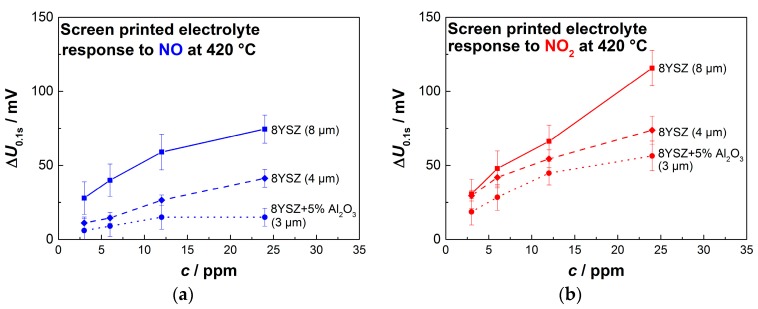
Sensor responses for sensors based on 8YSZ electrolytes built by screen-printing and sintering with overlying platinum electrodes towards small concentration of (**a**) NO and (**b**) NO_2_.

To summarize the sensor response of the screen-printed 8YSZ electrolytes, the sensor characteristics towards NO and NO_2_ are displayed in Figure 11.

Again, the concentration of NO and NO_2_ has a semi-logarithmic dependency on Δ*U*. However, 24 ppm of NO_2_ depart from that tendency in the case of 8–9 µm-thick electrolytes. The already mentioned decrease in sensitivity for the sensor with higher resistive electrolytes is clearly visible.

## 4. Conclusions and Outlook

Two different layouts of planar sensors that are operated by the pulsed polarization were investigated in terms of suitability as NO_x_ gas sensors. All sensors with buried platinum electrodes showed no response to any of the tested NO or NO_2_ concentrations between 3 ppm and 24 ppm, whereas overlying electrodes performed very well. This again underlines the importance of a well-designed three phase boundary between NO_x_ gas atmosphere, oxygen ion conducting 8YSZ electrolyte, and platinum electrode. When comparing sensors with porous, screen-printed electrolytes to dense films produced by aerosol deposition, lower noise and higher sensitivities towards NO, as well as NO_2_, are observed for the latter. A study with different electrolyte resistances of screen-printed and sintered electrolytes revealed that lower resistances are preferable for high NO_x_ sensitivities. In each case, sensor responses for NO_2_ were larger compared to NO.

An interesting finding is a decreased sensitivity towards NO when the sensor with interdigital platinum electrodes is operated only in unidirectional pulsed mode while still retaining its high NO_2_ sensor response. Further work on understanding the sensor mechanism is necessary to improve sensor responses. Also, cross-sensitivities to hydrocarbons should be reviewed for planar pulsed polarization sensors in the future.

## Figures and Tables

**Table 1:** Overview of all sensors with aerosol deposited (AD) and screen-printed (SP) 8YSZ electrolytes.

#	Composition	8YSZ Thickness	Position of Pt Electrode
AD 1	8YSZ	5 µm	overlying
AD 2	8YSZ	6 µm	buried
SP 1	8YSZ	8–9 µm ^1^	overlying
SP 2	8YSZ	4 µm	overlying
SP 3	8YSZ + 5% Al_2_O_3_	3 µm	overlying
SP 4	8YSZ + 10% Al_2_O_3_	3 µm	overlying
SP 5	8YSZ + 20% Al_2_O_3_	4 µm	overlying
SP 6	8YSZ	9 µm	buried
SP 7	8YSZ + 5% Al_2_O_3_	7 µm	buried
SP 8	8YSZ + 20% Al_2_O_3_	8 µm	buried

^1^ screen-printed two times to increase the electrolyte thickness.
